# Books Reviewed

**Published:** 1862-08

**Authors:** 


### EDITORIAL DEPARTMENT.

### BOOKS REVIEWED.

*Medico-Legal Contributions on Arsenic; containing Reports of a number of cases of Arsenical Poisoning, together with an account of the Methods employed in their Chemical Examination. By* CHARLES H. PORTER, M. D., *Professor of Chemistry and Medical Jurisprudence, Albany Medical College, Albany, N. Y. Albany:* CHARLES VAN BENTHUYSEN, 1862.

This pamphlet is a re-print of an article published in the transactions of the New York State Medical Society for 1862. It is, therefore, probably familiar to many of our readers. It is devoted mainly to the consideration of the methods of detecting arsenic, and the relation of a number of cases of arsenical poisoning, with the investigations had, most of which were conducted by the author. He, therefore, gives a record of his own experience, which entitles his remarks, in regard to methods of detecting arsenic, to greater weight. After a somewhat large experience, he says, that taking all things into consideration, the best method for the separation of organic matter from the poison, is that of Fresenius and Babo, though, in this, he differs from Mr. Taylor. Its merits are that, though its process refers mainly to the detection of arsenic, yet none of the metallic poisons would escape detection by it.

In this country, we all know the importance given to the chemical evidence in cases of poisoning; and probably without it, a conviction would never be obtained. Yet there are many fallacies to which the chemical evidence is liable. These the author very judiciously notices. The failure to detect a poison, upon examination, is not positive proof that the death was not caused by it. The same may be said of the presence of arsenic in the stomach, and the detection of minute quantities of it: for, in the former case, it may have been introduced after death, and, in the latter, it may have been derived from the chemicals used in the investigation. To obviate the one, some of the tissues of the body should be separately examined; and to prevent the other, great care should be taken to ascertain that the vessels and chemicals employed are free from poison. It is hardly necessary to speak of the errors likely to arise from entrusting the examination, in a case of suspected poisoning, to an ignorant or unskillful operator, for it is obvious that, in cases in which the chemical evidence is made so important, examinations should be made only by the most competent scientific men. Nothing short of accuracy should be allowed to weigh in a matter which may involve human life. We notice that the author considers Marsh's test for the presence of arsenic as more delicate than all others.

The cases to which the author's account of methods, is but a preface, and the relating of which is the main purpose of the publication, are, as before stated, those in which he has made the investigations. Among them, is the case of the People vs. Swan, which was tried in this city, and in which the interesting and conclusive experiments of Prof. Hadley, in relation to the effects of arsenic on apples, very satisfactorily established the innocence of the accused, and obtained his release after he had been convicted. This is the more interesting, from the fact that the well known scientific accuracy of Dr. Hadley was instrumental in establishing innocence of crime. The pamphlet is a valuable contribution to medico legal literature, and will well repay a careful examination.

---

*On Military and Camp Hospitals, and the Health of Troops in the Field; being the results of a Commission to Inspect the Sanitary Arrangements of the French Army, and incidentally of other armies in the Crimean War. By* L. BAUDENS, *Inspector and Member of the Council of Health of the French Armies, formerly Surgeon-in-Chief, and first Professor of the Perfecting School of Val de-Grace, etc., etc. Translated and Annotated by* FRANKLIN B. HOUGH, M. D., *late an Inspector of the U. S. Sanitary Commission. New York:* BAILLIERE BROTHERS, 440 *Broadway*, 1862.

The Medical Topography of the Crimea, is given in the first chapter and a description of the camps, hospitals, and many other circumstances and conditions of the army are included, together with the geological features of that country. It constitutes a very entertaining and instructive section.

The second chapter is upon rations, and a very minute and reliable account is given of the character, amount and cost, of soldiers food.

Chapter 3d—*Camps and Shelters.*—Contains a full description of the construction, location, drainage, ventilation, and other hygienic conditions of the camps, shelters and hospitals.

Chapter 4th—*Cloths.*—This matter of cloths is a very important one, not only in the view of the soldier, but also in the estimation of the Sanitary Commissioner; many valuable suggestions are made upon the subject of dress.

Part II, is upon *Infirmaries* and *field* hospitals, surgical operations, physicians, chloroform, &c., &c.

Part III, is devoted to hospitals and their diseases, cholera, typus, &c.,

&c., in the Crimea, to which is added an appendix, containing many valuable statistics.

This is one of the most readable books we have seen, telling the physician everything he would most desire to know about the Crimean war, and the results of surgical military practice everywhere. It is so attractive and easy in style, that intelligent men of all classes would be greatly interested in it; much of its teaching would be as useful to the military officer as to the surgeon. It is full of suggestions upon the whole subject of military science, though the main facts and observations have reference to the medical provision and treatment of the army.

It is a jewel of a book, and while we earneslty recommend it to the perusal of physicians, we also bespeak for it, consideration, as an open fountain of experience and observation, from which all my draw instruction and entertainment.

---

*Advice to a Mother on the Management of her Offspring. By* PYE HENRY CHAVASSE, *Fellow of the Royal College of Surgeons of England; formerly President of Queen's College Medico-Chirurgical Society Birmingham; author of "Advice to a Wife on the Management of her own Health." Reprinted from the Sixth London Edition. New York:* BAILLIERE BROTHERS, *Publishers*, 440 *Broadway*, 1862.

This book contains very valuable advice to mothers upon the general management of children in infancy, childhood, and youth. All the little attentions required in infancy are explained and full directions given, making the book a very desirable guide, to young mothers, which if possessed by them, would prove an excellent advisor, when otherwise some old, worn out, superstitious neighbor would be consulted.

Part II. contains chapters upon ablution, clothing, diet, exercise, amusements, education, sleep, second dentition and its diseases, and other topics applicable to childhood and its diseases.

Part III, is upon the care and management, education and training, of youth.

The book is really a very desirable one for a mother to read, and would protect many a family from imposition, besides, giving it truthful ideas upon the various topics discussed, in place of the absurd and irrational notions which are often entertained.

We have donated this little volume to our nursery library, and shall be happy to know that many of our friends have done likewise.

*The American Journal of Ophthalmology.—Vol.* 1, *No.* 1. JULIUS HOMBERGER, M. D., *Editor and Proprietor*, 24 *West* 12*th Street, New York. New York, July*, 1862: BAILLIERE BROTHERS, 440 *Broadway. Price,* $2 *per annum, in advance.*

The first number of this journal speaks favorably for its future success and usefulness, and we admire the enterprise which has prompted this undertaking. We bespeak for the *American Journal of Ophthalmology*, a just appreciation and generous support, by the profession.

